# 

**Published:** 1882-04

**Authors:** 


					﻿Whole Number,
CCXXXVII.
APRIL, 1882.
Number 9,
Volume XXI.
THE
BUFFALO
Medical and Surgical Journal.
EDITORS :
THOS. I.OTHROP, M.
P. W. VAN PEYMA, M.
A. R. DAVIDSON, M.
I.ITCIHN HOWE, M.
HERMAN MYNTER, M. ».
BUFF JL jUO :
Published by the Medical Journal Association.
Read the Notice of this Journal among the Advertisements.
Entered at the Post-Office at Buffalo, N. Y., as Second-Class Mail Matter.
				

